# Risk Factors of Recurrence and Malignant Transformation of Sinonasal Inverted Papilloma

**DOI:** 10.1155/2017/9195163

**Published:** 2017-11-09

**Authors:** Marta Gamrot-Wrzoł, Paweł Sowa, Grażyna Lisowska, Wojciech Ścierski, Maciej Misiołek

**Affiliations:** Clinical Department of Otorhinolaryngology and Laryngological Oncology, School of Medicine with the Division of Dentistry in Zabrze, Medical University of Silesia, Katowice, Poland

## Abstract

Sinonasal inverted papilloma is a relatively rare disease; however, it is prevalent enough for every ENT practitioner to encounter it several times throughout medical routines. Despite the developments in experimental and clinical medicine as well as surgical techniques, our knowledge of this disease is still inadequate. With improved imaging and better diagnostic techniques, proper diagnosis and qualification for surgical approaches leave no doubt. Although the endoscopic approach seems to be the gold standard for such condition, some cases may additionally require an external approach. Regardless of the type of surgery, postoperative management is crucial for both healing and long-term follow-up. Unfortunately, the procedures are still lacking in explicit and standardized postoperative management guidelines. Moreover, an important issue is still the need for a biomarker indicative of inverted papilloma and its malignant transformation. Several particles, within the spotlight of the researchers, have been SCCA, Ki-67, Bcl-2, Wnt proteins, and many more. Nevertheless, the topic requires further investigations.

## 1. Introduction

Sinonasal inverted papilloma (IP) was first described in 1854 by Ward and belongs to a rare group of Schneiderian papillomas—benign tumors deriving from the Schneiderian membrane—the embryonal ectodermal remnant forming the boundary between the nasal and sinus mucosa [1, 2]. It may differentiate into three types: oncocytic papilloma, fungiform papilloma, and, the most prevalent, inverted papilloma. The inverted papilloma is three times more frequent in men than in women and the peak onsets are recorded in the 4th–7th decade of life [2]. The location is usually unilateral with the origin at the nasal sidewall in the area of the middle nasal concha. Such tumors are characterized by rapid growth and lead to destruction of the osseous boundaries of the nasal cavity and the sinuses, spreading over the adjacent regions of the facial skeleton and the anterior cranial fossa [3]. The literature also described some single cases of ectopic location of such tumors (e.g., in the middle ear, pharynx, nasopharynx, oral cavity, palatine tonsil, and lacrimal sac) beyond the sinonasal mucosa which may be associated with displacement of the Schneiderian membrane during the embryonal growth [4, 5]. Etiology of the IP has not been well recognized. Mentioned among some potential factors having a role in respiratory tract remodeling into the inverted papilloma are, among others, chronic inflammatory condition, nicotinism, and HPV infection [5, 6]. The symptoms of IP include unilateral (most frequent) occlusion, relapsing nasal hemorrhage, and impaired sense of smell or anosmia. Such primary manifestations may be accompanied by headaches, lacrimation, or impaired vision [7].

## 2. Diagnostics and Treatment

**Figure 1:**
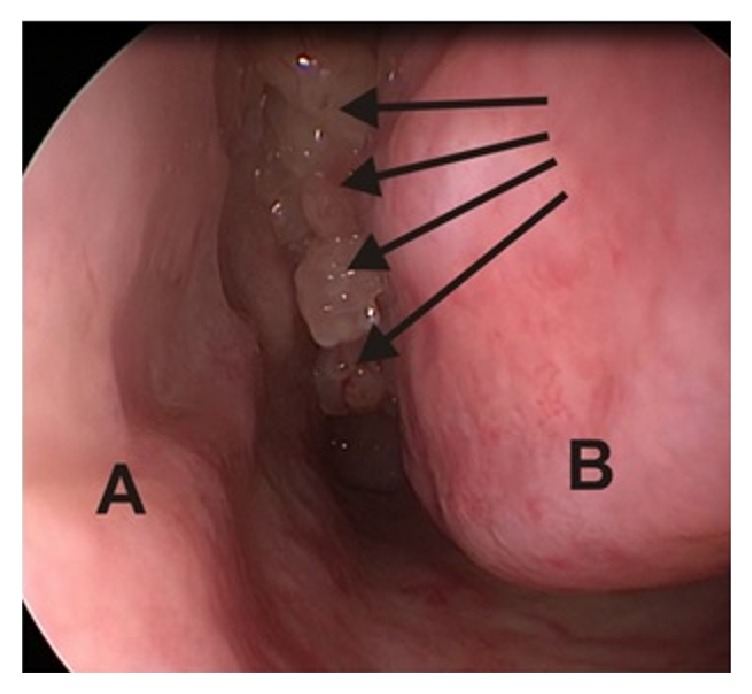
Endoscopic image of inverted papilloma in the left nasal cavity. (A: nasal septum; B: left inferior nasal concha; the arrows indicate the inverted papilloma masses.).

**Figure 2:**
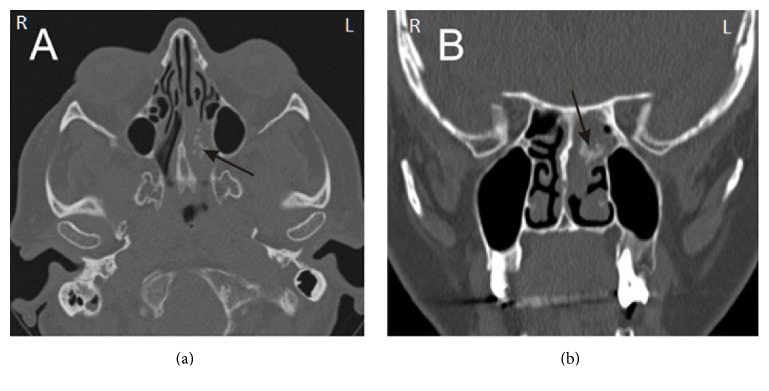
CT scan along the transverse (a) and frontal (b) plane in a patient with inverted papilloma of the nasal cavity and ethmoid cells at the left side; the arrows indicate focal hyperostosis.

Obviously, the diagnosis of IP needs histopathological verification. It is usually possible to take the material with the use of nasal and/or sinus endoscopy. The procedure allows clear imaging of IP as a pink/grey/brown polypous, soft tumor with nontransparent and uneven surface [2, 8] (Figure 1). Depending on location, it may be necessary to take a specimen through external access (extremely rare); it should be remembered, however, that an open biopsy brings about a risk of dissemination of the lesion over the adjacent healthy tissues [8]. Both computer tomography (CT scanning) and magnetic resonance (MR imaging) are elective investigations to evaluate the tumor extent and to design the method of IP surgery, although, because of bony structures visualization, CT seems more interesting for some surgeons [3, 9]. It is possible to locate the papilloma attachment site; in the CT scan, it is represented by a limited (focal) hyperostosis [10] (Figure 2).

**Figure 3:**
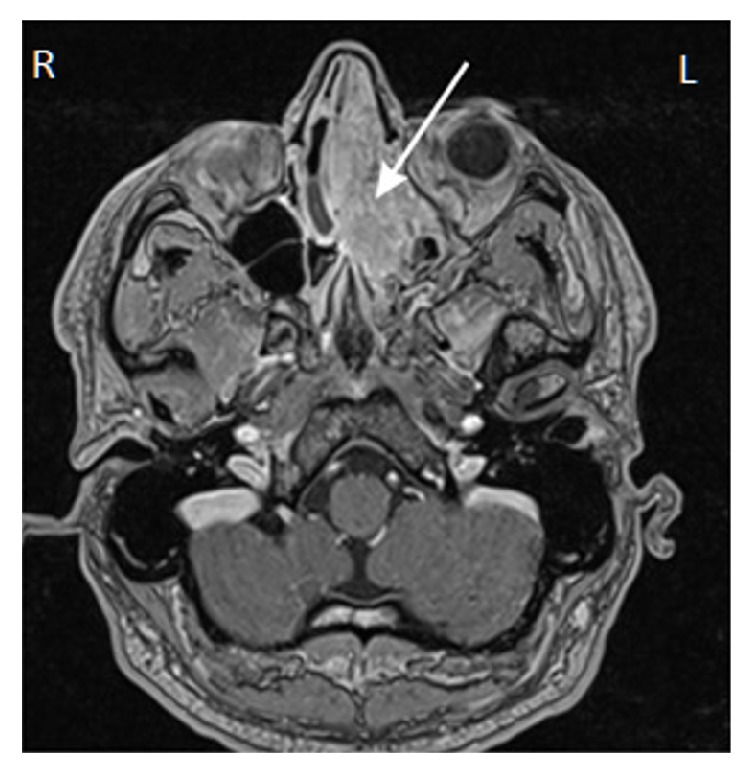
MR image along the transverse plane in a patient with left side (arrow) sinonasal inverted papilloma with visible tumor mass compression of the opposite side.

CT scanning does not allow, however, for explicit differentiation between soft tissues and secretions retained within the sinuses, which may result in overinterpretation of the extent of the lesion. In order to ensure an appropriate surgical method, it is suggested to perform complementary, preoperative nasal endoscopy or, if the tumor extends beyond the endoscopic range (frontal sinus, orbital fossa, and cerebral cranium), to complement the investigations with contrast-enhanced MR (Figure 3) and design additional external access. The intraoperative endoscopic image will ultimately verify the extent of the tumor, in most cases allowing for differentiation of the papilloma mass form the surrounding inflammatory lesions [3].

The most frequent method used currently to evaluate the clinical preoperative grade is the four-stage Krouse classification, taking into account the tumor location and malignancy [35]:

  T1: tumor restricted to the nasal cavity only
  T2: tumor restricted to ethmoid cells and/or medial wall of the maxillary sinus
  T3: tumor extending to the lateral, inferior, superior, anterior, or posterior wall of the maxillary sinus or penetrating the sphenoid or frontal sinus
  T4: tumor extending beyond the sinonasal boundaries and any malignant tumor

In 2007, Cannady et al. published the newest staging method showing correlation between anatomical extension and recurrence rate for IP managed by advanced endoscopic techniques [11] (Table 1).

The elective procedure for IP is total resection of the tumor [36]. The surgical treatment strategies were changing through the years. For a long time, medial maxillectomy with ethmoidectomy via lateral rhinotomy was recommended as the treatment gold standard for IP [5]. The situation has radically changed along with the progress in endoscopic solutions. The functional endoscopic sinus surgery (FESS), which is a minimally invasive endoscopic procedure to open the nasal sinuses, has been considered a gold standard for inflammatory conditions of the nasal sinuses for more than 40 years now [37]. Soon after the first endoscopic removal of sinonasal IP, described by Stammberger in 1981, endoscopic sinus surgery (ESS) became the method of choice for inverted papilloma and other tumors' management [38]. According to the Krouse staging, patients with stage T1 and T2 tumors may usually undergo successful endoscopic sinonasal surgery, while at stage T3 or T4 it is most frequently needed to design treatment assuming multiple transnasal and/or external access [39, 40].

The key issue is qualification of the patient for a proper method of the surgery which is supposed to remove a lesion radically, with special regard to the ossified foci [41]. ESS has numerous advantages, as compared to open procedures: no external traces of the surgery (scars or any other facial deformations of the patient), shorter healing, shorter hospitalization, and consequently reduced inpatient costs. Good intraoperative visualization facilitates resection of the tumor, preserving the healthy sinus mucosa and other anatomic structures and ensuring physiological functions of the mucosa to maintain proper olfaction and ventilation of nasal cavities and nasal sinuses. Moreover, it is always possible to supplement the procedures with external access. What is extremely important is that the observed recurrence rate following ESS was significantly lower than in case of open techniques [7]. It should be noted, however, that endoscopic techniques are extremely valuable only if performed by experienced surgeons who must be ready to supplement the procedures with external access, if needed [7, 36].

## 3. Recurrence and Malignant Transformation Risk Factors

The major cause for recurrence of the disease, or rather failure to cure it, is in case of IP the incomplete resection of the tumor or insufficient cleanliness of the surgical margin [40]. The risk depends, first of all, on proper selection of the surgery technique, as the highest rate of recurrence was observed following the restricted procedures, like polypectomy, traditional ethmoidectomy, sphenoidectomy, or Caldwell-Luc surgery, reaching even 80% [3, 42]. In one of the latest publications, Nygren et al. quoted the overall IP recurrence rate at the level of 25.3% with 9% malignant transformation, while the lowest recurrence rate was observed after combined treatment: endoscopic and open method [43]. The vast majority of the authors believe that, due to subsequent modifications of ESS, the risk of recurrence may even be as low as about 3%, confirming that the technique should be accepted as an elective procedure for IP [44, 45].

The most frequent recurrence is then in tumors located in sites with difficult access, such as the frontal sinus or even the maxillary sinus, the inferior wall of which may be found far below the level of the nasal cavity floor, where radical resection of a lesion in the region of* angulus anteromedialis* may appear impossible, if only via endoscopic access [15, 40, 46].

A condition for appropriate (radical) surgery is to resect the tumor along with the periosteum, with special regard to the ossified focus observed upon CT scanning, that is, a potential “origin site” of the tumor [3, 38]. Moreover, to ensure cleanliness of the surgical margin, it is recommended to resect the sinus mucosa and periosteum with 5 mm margin [40]. A standard procedure should be the intraoperative histopathological investigation to prove tumor resection with a margin of healthy tissues [46]. Nevertheless, one should not forget that IP recurrence may occur also in locations distant from the primary or at the opposite side, which may be explained by the theory of multicenter origin of IP [40]. The relation between IP stage, as per Krouse, and the recurrence rate is disputable. The available literature suggests more frequent recurrence in patients with higher Krouse stage tumor (mainly T3 and T4); however, the difference is not significant enough to hypothesize that ultimately [40]. For the time being, the literature quotes no uniform standard for postoperative procedures in IP. Early postoperative ENT follow-up aims at supervision of postsurgical healing, while long-term observation is intended to detect any possible relapse or malignant transformation early enough [40].

Based on our own experience as well as the congress contributions (14th International Rhinologic Conference, Rhinoforum 2015), our center adopted a postoperative procedure, assuming follow-up every three months during the first two years after the surgery, every six months during the subsequent two years, and then once a year for at least five years, as approx. 90% recur during that very period [47]. Moreover, a lifelong follow-up is recommended so that no later recurrence or metachronous foci are missed [41]. Endoscopic examination is a gold standard in postoperative follow-up in IP, complemented, if needed, with HP specimens or CT scanning and/or MRI, where suspected recurrence recommends MRI as a basic examination [47].

It is thought that about 9% of inverted papillomas transform into malignant tumors. The most frequent malignant tumor deriving from IP is squamous cell carcinoma [43]. A malignant tumor usually develops in the IP primary mass (synchronous focus); however, in some cases, it follows the surgical therapies of IP (metachronous focus) [2, 43]. Prognosis in squamous carcinoma in IP is poor. 5- and 10-year survival reaches 39.6% and 31.8%, respectively. The prognosis is poorer in tumors diagnosed in elderly age, infiltrating the cranial basis or the orbital cavity as well as being well advanced and showing low differentiation [48].

Lately, it was reported that MRI examination can be helpful in recognizing malignant transformation of IP. We already know that IPs in the conventional MRI should be indicated by a convoluted cerebriform pattern (CCP), a band-like region of hyperintense and hypointense signals on T2-weighted images or/and postcontrast-enhanced T1-weighted images. Recently, authors suggested that the focal loss of a CCP might be indicatory for IPs concomitant with malignancy [49, 50]. According to Wang et al., nonenhanced and static combined with dynamic contrast-enhanced MRI could be a useful tool for differential diagnosis of malignancy in IP [51].

The literature available contains scarce information on the recurrence risk factors in IP (Table 2) while the results remain unclear. Although no direct relation has been shown between smoking and the tendency towards malignant transformation in IP, higher tendency for IP recurrence has been observed in smokers. Resulting in swelling and chronic inflammatory condition of the nasal and sinus mucosa, smoking may contribute to elevated risk of recurrence following the surgery [52].

Although infection with the human papilloma virus (HPV) seems to show a documented relation with the onset of IP, the actual dependencies between HPV and pathophysiology of IP remain unclear, while the reports published are controversial [14, 18]. HPV infection is thought to have a role in tumor genesis through the viral oncoproteins E6 and E7. They disturb the cellular cycle mechanisms, which results in unregulated cell proliferation and oncogenesis, while the viral protein E5 enhances activation of the epidermal growth factor receptor (EGFR), leading to stronger mitogenic activity [27, 28, 53]. Recently, relation between HPV, in particular HPV-18, and higher risk of malignant transformation of IP has been emphasized again [54].

Restricted access, preventing accurate evaluation of the nasal sinuses, and difficulties in distinguishing an inflammatory condition from the papilloma mass upon imaging (both in the primary disease and during the follow-up of recurrence) indicate the need for an independent marker to establish explicitly the presence of IP itself as well as possible transformation into a malignant tumor.

Among the first IP associated markers evaluated and best described is the squamous cell carcinoma antigen (SCCA). This protein belongs to the group of serine protease inhibitors, serpins. SCCA is known as a free circulatory antigen, passively released into circulation by the squamous cells [55]. SCCA protein is a nonspecific marker, which has already found its use in monitoring and treatment of head and neck tumors, ovarian cancer, and lung or hepatic cancer [56]. It has been observed throughout the recent years that patients with IP show elevated SCCA in both IP diagnosed de novo and recurrence. Moreover, SCCA level was reduced in all patients after the surgery and no elevation was observed in sinus inflammatory conditions [30, 31, 33]. In some patients, circulatory SCCA was growing even before it was possible to observe a tumor macroscopically [30]. Also, the size of a tumor correlated positively with SCCA levels in IP. Some recent studies pointed to smoking as potentially influencing the SCCA levels; however, such relation needs further investigations [31]. What appears extremely interesting is that SCCA may also have a role in monitoring of IP transformation into squamous cell carcinoma. So far, two subtypes of this protein have been recognized, coded by different genes and showing slightly different structure and effect: SCCA1 and SCCA2 [32, 57]. Higher mRNA expressions of SCCA1 and SCCA2 were observed in the IP patients compared to individuals with carcinoma and inflammatory conditions [31, 58]. This very relation of SCCA2/SCCA1 may be used to detect squamous cell carcinoma in IP patients, as in the group of patients with malignant transformation it was substantially higher than in patients with IP only or the inflammatory conditions [31].

Other particles, whose role is still investigated and which may potentially have a prognostic value, are Ki-67, survivin, Bcl-2, Wnt proteins, metallothionein 2A, CCAAT, C/EBPs, C/EBP*α*, and CK10 proteins, E-cadherins, and *β*-catenin, as well as p16, p53, EGFR proteins, cyclin D1, and PLUNC (palate, lung, and nasal epithelium clone protein) (Table 3).

## 4. Conclusion

Sinonasal inverted papilloma is a statistically rare condition; however, it is prevalent enough for each ENT practitioner to encounter this disease multiple times throughout his professional routines. Although progress in experimental and clinical medicine and development of endoscopic surgical techniques extended our knowledge of IP, the treatment, diagnostics, and postoperative management demand further improvement along with better recognition of IP pathophysiology. Regardless of the surgical method selected, the* follow-up* must be based upon frequent visits with accurate endoscopic examination and, if necessary, additional imaging and microscopic procedures.

## Figures and Tables

**Table 1:** The Cannady classification [11].

	Localization	Recurrence rate
A	Nasal cavity, ethmoid sinus, medial wall of the maxillary sinus	3.0%
B	Other (than medial) walls of maxillary sinus, sphenoid sinus, frontal sinus	19.8%
C	Extrasinus extension	35.3%

**Table 2:** Factors elevating the risk of recurrence of inverted papilloma, as quoted by the literature available.

Factor described	Publications
Young age of patients	Jardine et al., 2000 [12]
Female sex	Suh et al., 1977 [13]
Tobacco smoking	Roh et al., 2016 [14] Jardine et al., 2000 [12]
Histopathological	
(i) Enhanced hyperkeratosis	Katori et al., 2006 [15]
and presence of squamous hyperplasia	
(ii) Elevated mitotic index	
(iii) Lack of inflamed polyps	
(iv) Greater amount of aneuploid cells	Liu, 1990 [16]

**Table 3:** Review of the literature on particles which may be potential biomarkers in the diagnosis of IP, recurrence, and malignant transformation.

Particles evaluated	Potential role	Literature
Antigen Ki-67, survivin, Bcl-2	Elevated expression of antigen Ki-67 as a marker of malignant transformation of IP	Tsou et al., 2014 [17]
	Ki-67 may reflect activity of the tumor cells' proliferation and may be used to measure IP proliferation rate	Meng et al., 2014 [18]
	Higher expression of Ki-67 antigen in IP than in an inflammatory condition	Mumbuc et al., 2007 [19]
	Positive correlation of nuclear (noncytoplasmic) immunoexpression of survivin and antigen Ki-67 and oncoprotein Bcl-2 in both evaluated tumors: IP and SCC	Stasikowska-Kanicka et al., 2013 [20]
	Immunoexpression of survivin, antigen Ki-67, and oncoprotein Bcl-2 was substantially higher in SCC than in IP and the controls	Stasikowska-Kanicka et al., 2013 [20] Lu et al., 2014 [21]
	Nuclear survivin and immunoexpression of antigen Ki-67 were substantially higher in IP group as compared to the controls	Stasikowska-Kanicka et al., 2013 [20]

Wnt proteins	Proteins of Wnt pathway, such as beta-catenin, cyclin D1, and Dvl-1 may have a key role in IP malignancy. Their levels correlate with IP stage	Jung et al., 2015 [22]

Metallothionein 2A	Protein involved in proliferation and infiltration mechanisms may be associated with pathogenesis of IP and also with increased local malignancy in IP	Starska et al., 2015 [23]

CCAAT or C/EBPs proteins	Significantly higher amount of C/EBP-alpha expressed upon IP recurrence than in primary tumor	Shabana et al., 2013 [24]

C/EBP*α* and CK10	They may be valuable markers of IP recurrence	Yaun et al., 2015 [25]

E-Cadherin and *β*-catenin	Markers helpful in monitoring of IP transformation into a malignant tumor	Koo et al., 2011 [26]

Proteins p16, p53, EGFR, and cyclin D1	Markers potentially helpful in monitoring of IP patients	Lin et al., 2013 [27] Yamashita et al., 2015 [28] Chao and Fang, 2008 [29] Mumbuc et al., 2007 [19]

PLUNC	Elevated expression in IP with multiple recurrence	Tsou et al., 2014 [17]

SCCA	Elevated level of SCCA correlates with IP, which can be used postoperatively for early or hidden recurrence diagnosis	Matoušek et al., 2014 [30] Suzuki et al., 2012 [31] Yamashita et al., 2016 [32] van Zijl et al., 2017 [33] Yasumatsu et al., 2005 [34]

SCCA2/SCCA1	Can be helpful to detect SCC malignant transformation in IP patients	Suzuki et al., 2012 [31]
